# Towards text mining therapeutic change: A systematic review of text-based methods for Therapeutic Change Process Research

**DOI:** 10.1371/journal.pone.0225703

**Published:** 2019-12-05

**Authors:** Wouter Smink, Anneke M. Sools, Janneke M. van der Zwaan, Sytske Wiegersma, Bernard P. Veldkamp, Gerben J. Westerhof

**Affiliations:** 1 Department of Psychology, Health & Technology, University of Twente, Enschede, Overijssel, The Netherlands; 2 Department of Research Methodology, Measurement & Data Analysis, University of Twente, Enschede, Overijssel, The Netherlands; 3 Netherlands eScience Center (NWO), Amsterdam, Noord-Holland, The Netherlands; University Magna Graecia of Catanzaro, ITALY

## Abstract

Therapeutic Change Process Research (TCPR) connects within-therapeutic change processes to outcomes. The labour intensity of qualitative methods limit their use to small scale studies. Automated text-analyses (e.g. text mining) provide means for analysing large scale text patterns. We aimed to provide an overview of the frequently used qualitative text-based TCPR methods and assess the extent to which these methods are reliable and valid, and have potential for automation. We systematically reviewed PsycINFO, Scopus, and Web of Science to identify articles concerning change processes and text or language. We evaluated the reliability and validity based on replicability, the availability of code books, training data and inter-rater reliability, and evaluated the potential for automation based on the example- and rule-based approach. From 318 articles we identified four often used methods: Innovative Moments Coding Scheme, the Narrative Process Coding Scheme, Assimilation of Problematic Experiences Scale, and Conversation Analysis. The reliability and validity of the first three is sufficient to hold promise for automation. While some text features (content, grammar) lend themselves for automation through a rule-based approach, it should be possible to automate higher order constructs (e.g. schemas) when sufficient annotated data for an example-based approach are available.

## Introduction

Big data and automated analysis methods are nowadays so omnipresent that they change traditional scientific research practices. Publications investigating new possibilities that these methods bring forth appear almost on a daily basis in every scientific discipline, and the field of psychotherapy research is no exception [1]. The rising popularity of machine learning methods to automatically analyse large bodies of data or texts accelerates research not only by allowing new kinds of research questions to be answered, but also by re-establishing the relevance of known questions which require the analysis of (text) data.

As early as Freud’s talking cure, the importance of looking at language to understanding the therapeutic process has been recognized [2]. The idea that the verbal exchange between counsellor and client contains important ingredients of therapy fuelled psychotherapy research [3]. There is a long-standing tradition of studying the linguistic ‘products’ of therapy (e.g. homework exercises, diaries, transcripts) in order to understand therapeutic change. The underlying idea is that the assessment of natural language use can reveal the process and changes over the course of therapy [4]. Thus, counsellor and client transcripts could potentially be a direct observation of the therapy process [5–7]. From this perspective, the psychotherapeutic process is considered a highly structured form of interaction, of which many important aspects are of linguistic nature.

Text-based therapy research has mainly relied on manual coding and human interpretation [8]. With the rise in available therapeutic texts in this digital age, automated analysis is making its entry into therapy research. There is now a growing number of studies on automated screening and diagnosis [9–12], and interest in automated analysis of the therapy change process is also picking up [13]. In our view, automated analyses in this field did not yet reach full potential because there are privacy and ethical concerns with sharing data from clients and patients [14, 15], and because the field is pragmatically organized. This means that data driven approaches prevail (there are –of course– exceptions, see [6, 16, 17]), and that –for some methods– the availability of data for automated analysis determined the research questions and approaches, rather than these decisions are based on psychological theory and research.

We propose that human interpretation and computer-based automated analysis can benefit from each other, and each have their distinct function. The large body of existing theories, models and methods for text-based analyses developed for understanding therapeutic change are currently underutilized. Yet, we would argue that these theories, models and methods are crucial for generating meaningful questions for automated analysis, and for a meaningful interpretation of patterns detected by a computer. Vice versa, the idea is that computers can be trained to perform (at least part of) the very labour intensive work of coding large bodies of text. This would enable the testing of hypotheses at an unprecedented scale, which is difficult to do with many of the existing methods that assess therapeutic change processes [8].

Therefore, we have the ambition to align automated analyses with existing text-based methods for therapeutic change processes. A prerequisite of a well-founded, meaningful development of automated text analysis is an overview of the available qualitative methods developed for the purpose of understanding psychotherapeutic change. Towards that end, we present our systematic review of literature on relevant peer-reviewed, published text-based methods for studying therapy change. In the remainder of this introduction, we first describe the field of Therapy Change Process Research (TCPR) [2], followed by a description of what *text mining* is, and what text mining has to offer in the context of understanding therapeutic change processes. We conclude with a discussion of *rule*- and *example*-based approaches for text mining.

### Therapeutic Change Process Research

Over a third of the people in most countries report problems at some time in their life which meet criteria for diagnosis of one or more of the common types of mental disorder (WHO; [18]). For example in the Netherlands, mental health problems contribute to about a quarter of the losses in Dutch health-life years [19]. In light of these statistics, it is not surprising that more than a thousand different psychotherapies have been developed [20]. Hundreds of studies already demonstrated that professional treatment can help people change in desired ways [21]. To ensure that these therapies are supported by sufficient empirical evidence the APA adopted a resolution on the effectiveness of psychotherapy [22].

However, progress in psychotherapy research is not made by only demonstrating the effectiveness of a treatment. In spite of thousands of studies publishing the outcomes and effects of therapies [5, 23, 24], the most intriguing questions remain: why and how do treatments work for whom? Studies aimed at average effects at group level fail to understand vast individual differences in responsiveness to therapy [25–28]. Therefore, TCPR “aims to identify the mechanisms through which psychological treatments bring about positive and therapeutic change” [2].

TCPR is accordingly defined as “the scientific investigation of what occurs during psychotherapy, with regard to its clinical meaningfulness; in other words, it investigates the process through which clinically relevant changes occur within psychotherapy” [29] (p. 259). Various names and definitions are used throughout the literature [2]: *Change Process Research* (CPR) [8, 30], *Psychotherapy Process Research* (PPR) [7], and some of the early works simply refer to ‘*change*’ [31–33]. To emphasize that we are dealing with change resulting from therapy, we propose to describe change processes as *Therapeutic Change Process Research* (TCPR) [2]. We use the terms *therapy* and *therapeutic* synonymously with *psychotherapy* and *psychotherapeutic*. ‘Change’ in TCPR then refers to the (positive) improvement in the client that is the result of psychotherapy (i.e. psychotherapeutic change). Although it is conceivable that therapeutic interventions (also) have negative effects, we limit ourselves here to the positive and beneficial effects of therapy.

Greenberg –who formally defined [T]CPR in 1986– was, together with Carl Rogers in 1961, among the firsts to argue for the importance of understanding change. Since then, many different TCPR methods have been developed [8, 31, 34]. Like other psychological research methods, TCPR methods also vary in their reliance on forms of statistical inference [35]. In a rather broad definition, qualitative psychological methods mainly rely on the interpretation of natural language [32]. Contrasting are quantitative linguistic TCPR research methods, that in practice usually equate to forms of counting of words [36]. LIWC, pronounced as the English name *Luke*, appears to be one of the forefront of the quantitative methods; in our current work, we however focused on the qualitative approach. For a more complete overview of (the differences between) quantitative and qualitative methods, see [29] (p. 259).

Most examples of qualitative approaches adhere to the interpretative study of the natural language used in therapeutic interaction [5, 8, 37], and are based on the assumption that word use reflects various psychological processes and change mechanisms [38]. Others identified cognitive, affective, and sharing empathy in sequences of therapeutic talk [39].

Over time, qualitative and quantitative approaches to TCPR developed into rather independent and different communities of researchers [31, 34, 40]. By systematically reviewing the qualitative TCPR approaches, we intend to present the state-of-the art, allowing for more integration of the two approaches. Clearly, there is room for doing so: the recent increase in web-based interventions (there is a variety of different names for *online therapy methods*, see [41, 42]), like e-mail supported life-review interventions [43, 44], generate textual data directly, discarding the need for transcriptions [3, 45], omitting this labour-intensive process. Also, data of therapeutic sessions are nowadays more easily collected than ever [11, 46, 47].

Nevertheless, the increased availability of these data did not lead to a substantial increase or popularization of TCPR research in general [29]: all developments resulted in larger availability of data, although this does not also automatically result in larger *access* to datasets for research. Partly, this is because the privacy of respondents is protected by ethical protocols and strict legislation, which prohibits data sharing and making datasets publicly available for TCPR [14, 15]. Another reason –and one that we shall discuss in detail– is because “the technology for evaluating psychotherapy [for the qualitative field] has remained largely unchanged since Carl Rogers first published verbatim transcripts in the 1940s: sessions are recorded and then evaluated by human raters” [12]. Indeed, development of the automated research methods is –relatively– slow (in comparison to other fields in Psychology and Psychiatry [48]).

### Text mining therapeutic change

As some argue that the amount of textual data currently available makes human evaluation no longer a feasible, valid or reliable method given realistic time- and budget constraints [3, 49, 50], it should not come as a surprise that text mining methods appear to be on the rise in psychology [51]. Text mining refers to a general methodological framework that includes several automated methods to analyse large corpora of texts. Practically, text mining approaches in psychology include counting words, identifying topics, and coupling the terms to a domain-specific ontology [47]. As text mining combines techniques and methods from many disciplines –including linguistics, statistics, computer science, natural language processing (NLP), artificial intelligence, information retrieval and data mining– it is not surprising that terms referring to the automatic extraction of information from text are used interchangeably, such as *text mining* and *NLP* [52]. Therefore, text mining is broad umbrella term that refers to a general methodological framework that includes several automated methods to analyse large corpora of texts.

We recommend novel and aspiring practitioners of text mining the works of [53, 54], and [55]. We recommend aspiring text mining practitioners the NLTK library, which is available in the programming language Python, and has an extensive step-by-step manual written by [56], which can also be used by those with little to no familiarity to programming or Python. We recommend [51] to those especially interested in text mining TCPR.

It is possible to identify a framework of studies that model change processes similar to what we aim to achieve by combining text mining and TCPR. For our purpose, we distinguished these works as *theory*- and *data*-driven approaches. The most well-known automated theory-driven text analysis tools is perhaps LIWC [36]. This text analysis program counts words in psychologically meaningful categories, and because it relies on previous research and theory to establish the relevance of the word categories it is considered a theory-driven method. Empirical results using LIWC demonstrate its ability to detect meaning in a wide variety of experimental settings, such as showing attentional focus, emotionality, social relationships, thinking styles, and individual differences [57].

Data-driven techniques are often developed to be broadly applicable, and regularly apply standard text mining tools to data with less reliance on a specific text analysis theory developed for that field. An example of a data-driven method is topic modelling, which refers to the use of type of statistical to discover abstract topics occurring in a collection of documents [58]. For example, [59] used this technique to analyse transcripts of therapy sessions from couples in a randomized trial, where the topic model establishes which words tend to occur together in transcript documents (e.g. *mom*, *mother*, *dad*, *sister*, and *brother* all belong the topic *family*).

The distinction between theory- and data-driven methods is then characterized by the extent to which methods incorporate theoretical knowledge. LIWC is a method that relies mainly on theory, whereas topic models are mainly data-driven. The characterization of theory- or data-driven methods becomes relevant in the context of what we would call the distinction between the *rule*- and *example*-based approach.

The former is based on annotated data and coding schemes, whereas the latter is based on linguistic information and text feature extraction. For example, [60] examined patterns across therapy by labelling utterances and sessions with several client-counsellor relationship variables. These manually labelled texts were then used for the text mining analyses. The rule-based approach is characterized by a more or less ‘automatic’ extraction of text features from texts through a set of pre-defined rules. For example, [61] determined several process verbs, and counted their occurrence in therapeutic sessions. Another example is [12], who rely on topic-modelling to cluster sessions based on similarities in word use in the in-therapeutic utterances.

Within the theory-driven approaches, we distinguish between those relying on rule- and example-based approaches. As we will argue, this differentiation within the text mining field is relevant to the question how TCPR research can (best) be automated. The distinction between theory- and data-driven methods is not made formally: the majority of methods is a hybrid, and –if one intends to classify methods based as theory- or data-driven– it is perhaps best to place methods a continuum where theory- and data-driven mark the edges.

### Rule- and example-based approaches

There are multiple approaches for text mining; we will discuss and highlight the importance of rule- and example-based approaches. Especially rule-based models are best understood in their historical context, but we keep the discussion of the history of the field to a minimum here (we refer the interested reader to [52]).

#### The rule-based approach

The earliest applications of what is now known as text mining come from computer scientists who –just after the Second World War– tried to model, analyse and understand speech and written natural language through rule-based language models [52, 62]. The work of these pioneers emphasized the core of rule-based models: some *input* (a text or verbatim) is mapped to an *output* (a label or a category) through some *function*. Rule-based language models describe a set of models that explicitly define the relation between input and output through a set of hand-coded rules for the function [63]. The rule-based approach thus mandates that the researcher explicitly specifies the routine by which lexical clues will be obtained, or that the researchers specifies exactly in advance which words contain relevant information.

For example, a comprehensive search string (‘regular expression’; [64, 65]) was used to detect whether an utterance contains a check question, suicide ideation, appreciation or surprise [66]. Similarly, decision trees with hand-crafted rules were used to classify sentences to open-ended questions [67]. The rule-based approach was also used to distinguish differences between linguistic measures and outcome measures was examined in high and low verbalized affect segments [61]. Others used the rule-based approach to show the correlation between verb repetition and differences in affective arousal [68]. This approach comes with the advantage that the researcher has direct control on what is extracted from the text. The other advantage is that theoretical knowledge can be directly applied: researchers often have a good idea on which words or expressions are related to their outcome of interest. The disadvantage is that when a researcher does not have theory to dictate what is important, it can be difficult to decide which words or information is are ‘more’ relevant than others.

Another disadvantage that limited the practical use of rule-based models is that the number of rules necessary to model natural language needs to be extremely large. Over the years, scientists from different fields (such as computer science and electrical engineering [52]) began experimenting with language models that were not based on comprehensive sets of rules, but that ‘learned’ to model language based on ‘raw’ examples from texts. Around 1990, this led to what many refer to now as a ‘statistical revolution’ [69]; example-based (machine learning) models became more prominently featured in text mining than rule-based models [55, 62].

#### The example-based approach

Around the 90s, computational resources and the availability of data both greatly increased (for example the large *Linguistics Data Consortium* became available [52, 70]), making way for example-based models, which typically demand more data and computational power than rule-based models. It turned out that the probabilistic data-driven models from statistics and machine learning were better suited for modelling natural language [71]. In about the span of a decade, example-based models completely took over the field [69].

To sharpen the contrast with rule-based models, we propose to call these models example-based, instead of ‘statistical’ or ‘machine learning’ models. The core of example-based models is that they rely on statistical inference to automatically learn the ‘rules’ of a language through the analysis of large corpora of typical real-world examples (instead of through specific hand-written rules [72]). More formally: the function is ‘learned’ by providing an exampled-based algorithm with specific examples of how the input and output should be associated.

The example-based approach is characterized by the application of text mining algorithms in order to find meaningful relations between human annotator derived labels (or ratings) and lexical cues in the data. The example-based approach mandates sufficiently large hand-coded datasets where differences in the text are related to differences in the outcome data [50]. For example, language models trained on (i.e. ‘machine learning’) hand-labelled counsellor utterances for low and high empathy sessions are used to predict empathy in sessions [73]. Annotated data are also used to automatically distinguish ‘change’ and ‘sustain talk’ in the client and counsellor utterances in motivational interviewing [9]. The practice is clear: without specification of any formal rule (that characterizes the rule-based approach), the example-based approach is able to learn, classify and predict labels with satisfactory accuracy if sufficient hand-coded data is available.

This approach comes with two drawbacks for the psychological practice. First, it requires a lot of hand coded data, which is not always available (because data sharing is not always allowed under strict privacy regulation [14, 15]). Second, the construction of such datasets is extremely expensive in both annotator-hours and cost [49]. Since the performance of many natural language processing tasks is limited by the amount and quality of data available to them [74], one promising alternative for some tasks is the rule-based approach.

**Fig 1:**
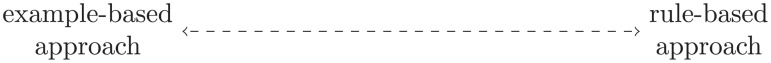
Automation approaches. Research methods in Therapeutic Change Process Research (TPCR) can automated based on the extent by which they rely on a rule- or an example-based approach for automation.

Note that our distinction between example- and rule-based approaches does not mean that these two approaches are mutually exclusive. Fig 1 reflects our view on the matter: the two approaches form the ends on a spectrum. A method can rely on both approaches for automation, but usually one of the two can be preferred over the other when a first attempt is made at automation.

### Research goals

TCPR aims to connect in-therapy change processes to outcomes. Qualitative instruments are commonly used to study the linguistic products of therapy. However, due to the dependence on human interpretation these methods are limited in analysing the large bodies of text that are nowadays available limiting their use to small scale research. We therefore advocate the combination of TCPR and text mining. Towards that end, we present a systematic review in which we aim to provide an overview of the commonly used methods, peer-reviewed qualitative text-based TCPR methods, assess to what extent these methods reliable and valid, and assess the extent to which these methods are automatable based on the rule- or example-based approach.

## Method

**Fig 2:**
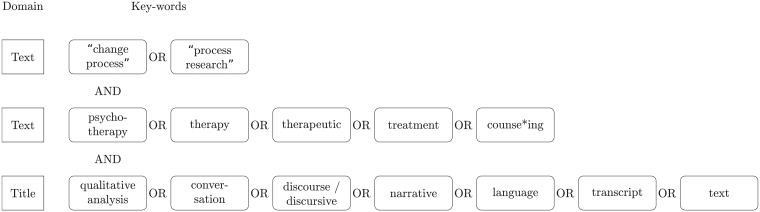
Search query. Search query used to search the PsycINFO, Web of Science and Scopus databases. The key-words have blocks with round corners; blocks with sharp corners indicate the search query’s domain (assessment of the title, or the full-text). * indicates the use of a wild card, when different forms of spelling can be used. / indicates that two words are treated as equivalents.

A commonality of TCPR is the frequent co-occurrence of ‘process research’ and ‘change process’. We expressed interest in psychological treatments through the queries ‘psychotherapy’, ‘counselling’, and ‘treatment’. We identified qualitative TCPR through the queries ‘language’, ‘text’ and ‘transcripts’, including ‘narrative’, ‘discourse’ or ‘conversation’ analysis, see Fig 2 for an overview of our search query.

We used the first and third block of Fig 2 to search titles, abstracts and full-texts, the second block was used to search only titles. As treatment-related queries frequent in all psychotherapy articles, inclusion of these terms for full-text searches led to an increase of many articles not directly related to the research question. To ensure good coverage, we included several important and impactful TCPR publications, for which we consulted TCPR experts.

### Databases

We searched three scientific databases: *PsycINFO*, *Scopus*, and *Web of Science*. PsycINFO should contain many TCPR records as it centres on *psychology*, the *behavioural* and the *social sciences*. We also used Scopus, as it contains the *MEDLINE* databases, which also span *psychiatry* and *medical psychology*. To also include the *humanities*, we also searched Web of Science.

**Inclusion** The inclusion criterion is that a study has to report on a *TCPR instrument* through the *assessment of language* or *text-components*, such as transcripts, diaries, e-mails, psychotherapeutic assignments.

**Exclusion** Reasons for exclusion besides not meeting the inclusion criterion were: not a scientific publication (e.g. commissioned report, organizational project paper, book or book review); not an empirical study (e.g. theoretical perspective on change or therapy); aimed at another change process than therapy (e.g. career counselling, flourishing); not a target group with common mental health disorders (e.g. stuttering, sexual offenders HIV patients); and not measuring (individual) client-counsellor interactions (e.g. group-therapy, family-therapy).

### Identification and selection of methods and studies

After removing duplicate articles, the first and last author independently screened all titles and abstracts for inclusion and exclusion criteria. Identifying the articles to exclude turned out to be relatively straightforward. Agreement upon inclusion was not so easily reached, and we calculated Cohen’s *κ* < .70. This statistic mainly reflects that TCPR-related literature was addressed by multiple different disciplines under a variety of different names, making it difficult to reach agreement upon inclusion.

One of the two screeners was an experienced TCPR-researcher, the selection of this screener turned out to mark more articles for inclusion. To avoid the risk of excluding relevant articles –which is the largest risk when *κ* is below the cut-off point of .70– we decided to include all articles that either one of raters selected for inclusion. All literature marked for inclusion was then fully read. Both screeners labelled the articles with the method that the authors used. Finding the frequently used methods then –essentially– boiled down to counting all the methods that were found.

We chose to only give an elaborate description the methods that were mentioned more than twice in the literature. We made the assumption that the methods that were used only once or twice could not have had an lasting impact on the TCPR field.

### Data analysis

We assessed the full-texts of the articles that used frequently occurring methods in three steps, one for each of the three research questions. Data analysis in these three steps was conducted by the second author and checked by the first author. As basis for the analysis we identified one key article for each of the methods. The first article where the method was described in detail, or the article that was referred to by all other articles using that method, the ‘source’-article. This was supplemented by an analysis of articles citing the key article and/or using the same method. We choose the key article to be the article that first proposed the method, or contained the most information on how to specifically apply the method.

**Step 1. Description of the methods** Here we describe, mainly on the basis of the key article, if and how the theoretical background and main concepts of the method are provided by the authors of the method. We paid attention to how explicit and elaborated underlying theoretical models and concepts were described.

**Step 2. Assessment of quality criteria** We looked at the reliability and validity of the included methods. For our assessment, we first analysed if and how authors provided argumentation to explicitly address the validity and reliability of their method. This analysis of explicit accountability for the quality of the methods was complemented by our own analysis of more implicit evidence for the quality of the methods either within the key article, or by reference to other articles adopting the same method. For assessment of the validity of a method, we looked at *internal* and *external validity*.

We deemed a method *internally valid* to the extent that claims and constructs were substantiated with existing theories and models, and/or empirically validated using transcripts and examples. Internal validity increases to the extent that an underlying theoretical framework or model (for the method as a whole and/or for key constructs to be measured), is made explicit and detailed by authors. In anticipation of the question about the automatibility of the method, we additionally described whether applicability included the availability of linguistic markers for the identification of labels (this also added to the reliability of the measure).

The *external validity* of a method increased when transferral to other contexts, client groups, or therapies is made plausible. As indications for transferability of the method, we looked at explicit argumentation by the authors, and for evidence that the method has been used in various applications. In addition, we looked for more implicit indications for transferability, such as the provision of points of comparison which enable analogical reasoning necessary to discern commonalities and differences with other cases where the method could have been applied [75].

*Reliability*. We deemed a method reliable when the description of the method demonstrated *consistency* (the extent to which data can be analysed independent from other raters and arrive at the same conclusions) and *transparency* (the possibility to virtually replicate the procedures, failures, and successes of the original study). We assessed the consistency of a method based on the reporting of the inter-rater reliability score of the coding scheme (if provided), and the transparency of the method depending on the presence of a manual or coding system with good labels, examples of texts and a clear operationalization.

**Step 3. Assessment of automatibility** Because these qualitative methods were not originally meant for automation, we deduced the potential for automation from the combination of traditional criteria for methodological quality, e.g. reliability and validity. In our view, reliability is a necessary condition for a method to have potential for automation: if human raters cannot reach good reliability, automated methods cannot be expected to do better. While this may generally apply to all forms of text mining, we made a distinction between a rule- and example-based approach for text mining, to let the qualitative research practice better align with the nature of text mining methods.

An example-based approach to text mining requires the availability of a good coding scheme with high inter-rater reliability. Based on large amounts of manually coded data, a computer can be trained to repeat the analysis. The accuracy of the computer in analysing which text segments are associated with which codes, can then be tested using a test set (again consisting of annotated data). A rule-based approach to text mining on the other hand, does not require any manual coding, but rather depends on the availability of linguistic markers for TCPR-related constructs. The more information about word use, grammar, or other linguistic features form text are provided, the higher chances that a suitable text mining tool can be identified (or developed) for mining the construct.

## Results

**Fig 3:**
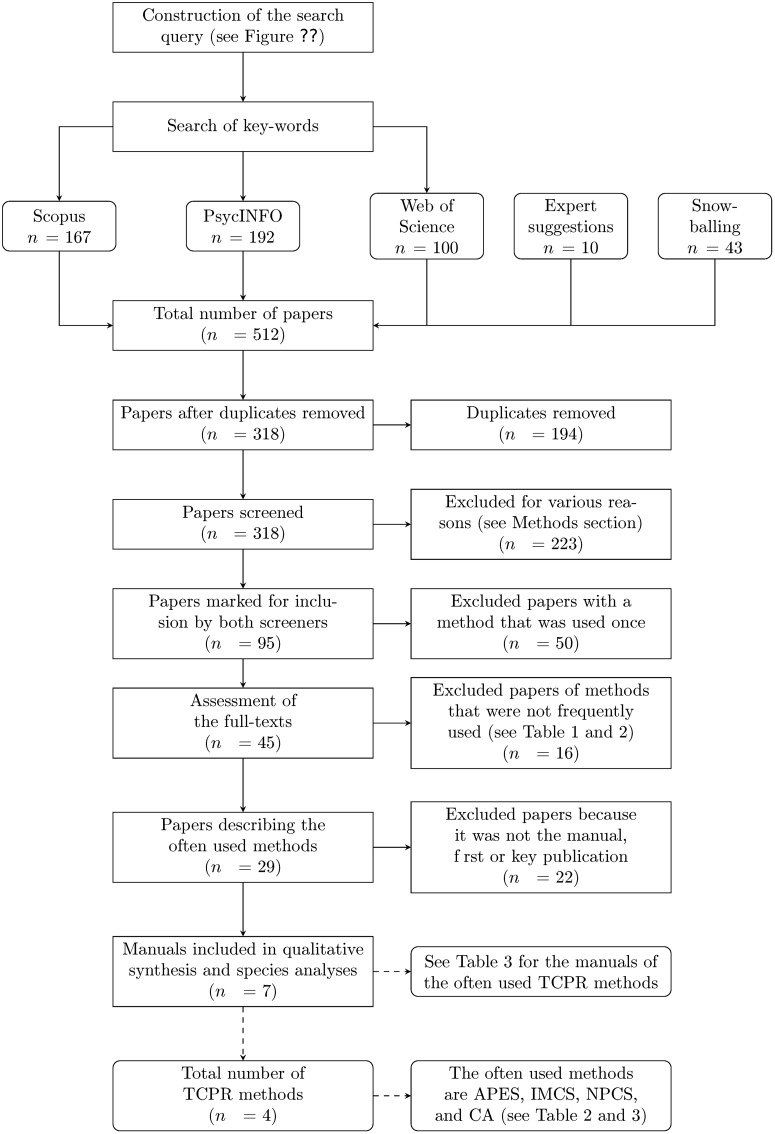
Flow of information. Flowchart of the information through different phases of the systematic review. In total, we included 7 articles describing 4 methods, see Table 3 for the abbreviations of the methods and the corresponding articles.

Our search resulted in 192 articles in Scopus, 167 in PsycINFO, and 100 in Web of Science, see Fig 3. Independently, both raters selected (in total) 95 unique articles. These 95 articles described a total of 62 methods that met the inclusion criteria in the opinion of either one or both of the authors, see Table 1. 80.6% of these methods were only mentioned once, covering 52.6% of all the included literature (percentages can be calculated from Table 1). The other 12 methods, which are described by 45 articles see Table 2 (and Fig 3), therefore also covered (slightly less than) half of the included literature, but are far more likely to have impacted the field. Eight of these methods were used only twice (see Tables 1 and 2); the other four methods occurred more than twice (see Table 2), and cover 64.4% of all methods that occur more than once (see Table 1). After reading the full-texts of the 29 articles describing the often used methods (see Fig 3), we entered *N* = 7 articles in our study (see Fig 3), describe *N* = 4 TCPR methods (see Fig 3).

**Table 1:** Number of articles that mention different TCPR methods.

	*Methods*	*Articles*
Once	50	50
Twice	8	16
Often used	4	29
*Total*	62	95

The 62 methods were disaggregated to whether they were mentioned once, twice, or more than twice in the literature. We assessed the full-texts of 45 articles (out of 95 in total, 47.4%) of methods that were mentioned twice or more often.

**Table 2:** Methods and how often they were encountered in the literature search.

#	*Methods*	*Abbr*.	*Count*
1	Innovative Moments	IMCS	16
2	Conversation Analysis	CA	5
3	Assimilation Analysis	APES	4
4	Narrative Process Coding Scheme	NPCS	4
5	Comprehensive Process Analysis		2
6	Core Conflictual Relationship Theme		2
7	Discourse Analysis		2
8	Metaphor Analysis		2
9	Return-to-the-problem markers		2
10	Structural Analysis of Social Behaviour		2
11	Thematic Analysis		2
12	Therapeutic Collaboration Coding Scheme		2

The first four methods are the methods that were used most often. We included these four methods in our review. In total, we found 29 articles describing the four often used methods, see Fig 3. We also included the abbreviations of their manuals and codebooks, see Table 3.

### Terminology

We included these four frequently used methods (by including their –in total– 7 manuals) in our review: *Assimilation of Problematic Experiences* (APES) [76, 77], *Innovative Moments Coding Scheme* (IMCS) [78, 79], *Narrative Process Coding Scheme* (NPCS) [80], and *Conversation Analysis* (CA) [81, 82], with IMCS clearly outranking the other three methods in terms of frequency (16 articles using this method as opposed to 5 times conversation analysis, and 4 times the other two methods, see Table 2).

We extensively studied 7 articles, describing 4 methods, see Table 3. The terminology that we used to denoted the methods and articles will be in similar fashion, to which we (sometimes) refer interchangeably. To avoid confusion, we explicitly described the methods and their manuals in Table 3.

**Table 3:** Manuals of the often used methods.

*Short*	*Scale*	*Manual*
IMCS	Innovative Moments	[78]
	Coding System	[79]
CA	Conversation	[82]
	Analysis	[81]
APES	Assimilation of Problematic	[76]
	Experiences Scales	[77]
NPCS	Narrative Process	[80]
	Coding System	

The seven manuals belong to the four methods mentioned in Table 2 and Fig 3.

### Coding schemes

We answer the research questions for each of these four methods. The first (description of background and main concepts) and second research question (assessment of their validity and reliability) culminate in the third question (potential for automation). This assessment was done by the first two authors. See Table 4 for an overview our findings on the quality assessment of these methods. We will discuss the content of Table 4 in detail in the four following sections, one for each method, starting the most often used methods (see Table 2). If training data is present, we checked whether a references was made to this training set (for example [79], refer to their training data).

**Table 4:** Overview of the quality of the frequently used methods.

*Method*	*External* *validity*	*Internal* *validity*	*Reliability*	*Annotated* *data*	*Linguistic*
IMCS	Medium	Medium	High	High	?
CA	Low	Low	Low	Low	Low
APES	High	High	Medium	Partly	Promising
NPCS	?	Medium	High	High	Promising

We abbreviated the four often used methods: *Assimilation of Problematic Experiences* (APES) [76, 77], *Innovative Moments Coding Scheme* (IMCS) [78, 79], *Narrative Process Coding Scheme* (NPCS) [80], and *Conversation Analysis* (CA) [81, 82]. “?” refers to *unknown*, or *not assessable in its current form*.

### Innovative Moments Coding Scheme

IMCS screens therapy sessions for innovative moments (IMs). IMs are defined as “episodes in which the person did, thought, imagined, or felt something different, or related to others in a new way, distinct from the rules that the problematic self-narrative ‘prescribes’ for his or her life” [78] (p. 107). An IM is a concept derived from narrative therapy. The underlying narrative approach to change is contrasted with traditional psychotherapeutic models (e.g. social rather than psychological, focused on re-authoring of alternative stories rather than repairing deficit). While starting out as an alternative to traditional therapeutic models, the method is proposed to have wide applicability even extending to the study of change in everyday life. The authors make their aim of generalizing the method to diverse psychotherapeutic models and different populations explicit. A further claim for the external validity of the method is presented by emphasizing its compatibility with mixed methods, including statistical procedures and analyses, see Table 4.

Five types of IMs are distinguished: action, reflection, protest, re-conceptualization, and performing change IMs. These five types are clearly defined and corroborated with literal excerpts. The methodological procedures for conducting IMCS involve data analysis by two raters who are blind with respect to the outcome (therapy success) of the cases under analysis. Rater training involves the identification of the on-set and off-set of IM as well as categorizing them into the five types. The authors developed a coding training protocol, which when followed successfully results in Cohen’s *κ* of at least .75. So, IMCS fulfills requirements for consistency and transparency.

Linguistic markers which can provide an avenue for the rule-based approach for the five categories are not clearly given in the codebook, but in the theoretical background description, some of the main concepts are discerned with a keen eye to grammar and word use, as can be seen in the distinction between an internal account of oneself (“*My problem is that I have a low motivation to study*”) and an intentional state formulation (“*My problem is that I feel that my parents want me to* …, or “*I’m not so sure that I want that* …”). The existence of a reliable coding scheme for the analysis of IMs makes this method suitable for the example-based approach. The presented linguistic markers for identity and agency related concepts show promise for translation into the rule-based approach, see Table 4.

### Conversation analysis

Under the umbrella term of CA, we found 5 articles that applied to this method to study TCPR, but –like *discourse analysis*– with great variations in its application. Although CA for TCPR tends to be used “more generally and descriptively at the conversation structure of therapy sessions rather than specifically focusing on the change process” [8] (p. 129), there are notable exceptions such as [81]. However, the method’s reliance on in-depth analysis of change sequences within a single case, together with its highly context-bound nature, contribute to low external validity. The authors’ own suggestion that the method can be viewed as a complement to other therapy change process methods such as assimilation analysis supports the impression that there is no specific theory or model guiding the method [81]. So, while the method seems to be a promising addition to TCPR by offering a method for understanding the interaction between counsellor and client, the internal validity of therapy change (the central concept), is low, see Table 4.

Regarding reliability, it should be noted that transparency is high in the sense that literal citations are presented with elaborate argumentation anchored in the text. We could not find one specific codebook, procedure or approach that several authors repeatedly used for TCPR purposes (see [81] for an overview of CA applied for psychotherapeutic research). Commonly, TCPR-researchers referred to [82], and [81], see Table 3. All in all, the low validity and reliability (at least concerning consistency) of CA, makes its suitability for the example- and rule-based approach highly unlikely in its current form. However, CA is more generally known as a rigorous method, here evidenced by high transparency and adherence to CA rigorous guidelines for anchoring analysis in actual text [82]. This characteristic of CA potentially renders it automatable using a rule-based approach to text mining, however, this potential is relatively low in comparisment to the other three methods, see Table 4.

### Assimilation of Problematic Experiences Scale

The APES method is –compared to the other three methods– based on the most integrated model of therapy change, and presents a generic model to study change that is common to all psychotherapies. Its external validity is substantiated by a wide range of concepts and phenomena from various therapeutic approaches as diverse as cognitive behavioural therapy and psychodynamic approaches. The central idea is that these diverse approaches share a common component: the assimilation of problematic experiences. As the experiences are assimilated, clients move through predictable stages. The generalizability of the assimilation concept is dealt with in a paragraph dedicated to its generality [76] (p. 416). That external validity is an explicit goal of this method, can also be seen its comparative objective of providing “a reference point for evaluating the effectiveness of alternative techniques” [76] (p. 411).

Reliability of the method is also mentioned from the outset of the article by emphasizing the aim of developing “a concise, internally consistent, researchable model”. This ambition is then made concrete in definitions of the main concepts (e.g. schema, problematic experience, assimilation and accommodation), and descriptions of the partly overlapping stages of assimilation (unwanted thoughts, awareness, problem clarification, insight, problem solution). The main concepts vary in the extent to which linguistic markers are specified to identify their occurrence in therapy text. For example “therapists and clients develop words or phrases that bring to mind a constellation of concepts or memories, that is, re-evoke useful schemata” is much more abstract than example phrases indicating problematic experience (e.g. “*I don’t know what this is about*”, “*this is not like me*”, or “*I can’t stand this about me*” [76], p. 412). Application of the method is illustrated using two session summaries, where in-session citations are used to link actual word use to overarching concepts (e.g. schemata). No session transcripts are provided. We found no public access to the mentioned codebook.

All in all, APES scores highest in terms of external and internal validity, but we could not fully assess its reliability due to lack of publicly available information about a code book or inter-rater reliability results. Therefore, we expect that automation through the example-based approach of APES is possible, providing that highly reliable manually coded data become available, see Table 4. The potential for a rule-based approach to APES is dependent on the abstraction level of the constructs to be mined. We would suggest that problem experiences are detectable by text mining methods and techniques on the basis of content and grammar, whereas identification of a higher order concept such as schema is much more difficult to automate.

### Narrative Process Coding Scheme

The main concepts in NPCS are narrative disclosure, emotional differentiation and reflexive meaning-making. These three concepts allow the identification of three process modes: storytelling, emotion, and meaning-making/reflection. The internal validity and coherence between these concepts is given explicit attention by the authors, who use the dialectical constructivist model to substantiate the presented coding scheme [83]. The application of this method is from the outset of this article presented as specific to emotion-focused therapy, although the underlying dialectical constructivist model of therapeutic change appears to have wider relevance to other kinds of therapy. Therefore, external validity in terms of generalizability of the method to various types of therapy does not appear to be an objective of the authors.

In contrast, reliability of the coding scheme is made an explicit point of attention throughout the article, starting with “a two-step procedure that enables researchers to reliably subdivide and characterize therapy session transcripts into topic segments” [84] (p. 90). Topic segments are defined by key issue and relational focus. These topic segments are then further divided into the three process modes. Identification of these modes requires interpretation of a text excerpt of “at least ten transcript lines in length” on the basis of three interpretive questions: storytelling refers to text addressing the question “*what happened to me*”, emotion to the question “what was felt by the client”, and reflection to “*what does it mean to me now*”. The authors report that “good levels of inter-rater agreement were established” at the level of topic segments as well as process mode. Literal citations of session excerpts are presented, yet no specific word use indicative of the three modes is mentioned. However, the authors mention that they have recently found problem markers (e.g. same old story, empty story), and change markers (e.g. unexpected outcome stories, healing stories). Notably, these markers are at the abstraction level of stories and not at the level of specific utterances.

In sum, we conclude that NPCS as presented in the key article, is in our view both internally valid and reliable, yet its external validity in terms of applicability beyond emotion-focused therapy of depression is unclear. We consider the feasibility of the example-based approach of NPCS high, because of the existence of a highly reliable coding scheme. Despite the lack of linguistic markers reported for the three modes (storytelling, emotion, reflection), we consider the rule-based approach possible, considering that the three modes correspond closely to some of the widely used LIWC categories (cognition, emotion).

## Discussion

Because half of the coding schemes we found were used only once or twice, we have come to the conclusion that TCPR is a fragmented field with few attempts for unifying the field. We included the seven manuals of the the four frequently used TCPR methods in our review: IMCS, NPCS, APES, and CA. The first three methods mainly focused on the improvement of the client, CA focused on the interaction between client and counsellor. CA has a low validity and reliability score, rendering both the example- and rule-based approach highly unlikely in its current form. Incidentally, the CA literature on psychotherapy is perhaps best considered as a specific application of CA, because CA is –primarily– a methodology for the analysis of social interaction, which has –on occasion– been applied to therapeutic data. We therefore do not include CA in the general reflection on the example- and rule-based approach that we give below. We refer the interested reader to [82] for a review on specific areas of CA and psychotherapy, to [81] for an example of how CA can be applied in a TCPR-setting, and to [85] for a discussion of CA and coding.

The other three methods are in its present state ready for automation to varying degrees, depending on whether an example- or rule-based approach is adopted. Both approaches require an investment for each of the three methods. If sufficient hand-coded data is available, the example-based approach would be very feasible for all three methods; if not, then researchers considering this approach should reflect on whether investing in (manually) annotated datasets outweighs the costs. The rule-based would then perhaps be more feasible.

### Example-based approach

We conclude that the existence of a reliable coding scheme for the correct classification of important moments makes the IMCS suitable for automation based on the example-based approach. For the APES, we estimate that example-based automation of APES is possible, providing that highly reliable manually coded data become available. We consider example-based feasibility of NPCS high, because of the existence of a highly reliable coding scheme.

### Rule-based approach

The presented linguistic markers for identity and agency related concepts of the IMCS show promise for translation into the rule-based approach. The potential for a rule-based approach to APES is dependent on the abstraction level of the constructs to be mined. Despite the lack of linguistic markers reported for the three modes (storytelling, emotion, reflection), we consider a rule-based approach possible, considering that the three modes correspond closely to some of the widely used LIWC categories (cognition, emotion).

### Strengths and limitations

We aimed to cover the whole TCPR field, but only intended to include the frequently used methods in our review. We started by developing a comprehensive search string, but ensured good coverage of all relevant publications of the TCPR field by also including suggestions of experts. As our systematic review was designed to let the most important and influential publications surface, we felt that we can indeed give an adequate description of the TCPR field.

We also devoted special care to development of our search string as the TCPR field appeared to be a fragmented field. We included queries that covered the psychological literature broadly, the TCPR literature specifically, and text-based methods exclusively. The combination of these queries were used such that we included the whole TCPR field in the initial phase of our review. Because our search string was able to produce the entries that were suggested by experts, we felt that our search strategy covered the literature sufficiently. We adopted the PRISMA standard to ensure that our search was reported transparent and completely.

To avoid placing emphasis on the lesser used methods we focused our study on the commonly used methods, which spanned half of the literature. In doing so, we feel that we included the methods that with a high to good reliability and validity. These quality criteria can only be established through frequent usage, which mandates a focus on the methods used often enough to rightfully claim sufficient reliability and validity. We also valued repeated use of the method particularly because re-use also indicates applicability to multiple data-types and is proof of external validity.

We noticed that the methods that we included mainly focused on the improvement of the client, rather than methods that were tailored to the counsellor. CA focused on the interaction between client and counsellor, but is in its current form not suitable for automated TCPR research. A reasons why we did not find such methods could be because we excluded scientific books from our search. However, as we reviewed the peer-reviewed literature extensively, publications using methods described in books should have surfaced.

It was our choice to exclude methods that were not often used. However, we would like to encourage the researcher interested in these excluded methods –or a specific method– to use our search string. Researchers interested in the (specific) active change processes itself (rather than the TCPR methods), should review the literature with search queries aimed specifically at these processes. From our search, we conclude that these processes do not arise from generic search queries aimed specifically at therapeutic change processes.

We chose to focus only on the text-based methods to study TCPR because we wanted to establish the potential of text mining. Hence, we focused specifically on texts and we did not include non-verbal and behavioural aspects. These aspects contribute to the therapeutic process; however, as they are not textual, they fell outside of the scope of our review. Explorations of these aspects is therefore open for other TCPR researchers.

Ethical and privacy concerns remain a practical issue for data sharing among TCPR researchers. To stimulate sharing, future research into high quality data anonymization software is now more important than ever. Another option could be for researchers to collaborate in national or international colloquia, that –under strict rules and in line with legislation and informed consent– allow for datasets to be shared internally.

### Final remarks

One of the intriguing questions of psychotherapy is how therapeutic processes bring about therapeutic change. As the amount of therapeutic texts is rising in this digital age, new computational methods and research questions became available. These methods enable the investigation of large bodies of therapeutic texts. To facilitate the transition of TCPR from small(er) to Big data, we discussed potential for automation of the most used TCPR methods.

We hope that our systematic review contributes to the unification of the TCPR field. Determining the commonly used methods helps both aspiring and seasoned TCPR researchers to get some perspective on the different research directions in TCPR. Investigation of the potential for automation (of these methods) could also help researchers to determine whether automation is an interesting research direction. We think that automation is important because the (automatic) detection of change processes in therapeutic texts can stimulate the *what works when for whom* discussion, which is not only relevant for psychotherapy researchers, but also for clinical practitioners.

Establishing the potential for automation through the example- or rule-based approach helps TCPR researchers to decide if their datasets are suitable for automation. It also aids in the consideration whether automation is worthwhile. Automated text-analysis methods could also bridge the quantitative and qualitative disciplines, which are sometimes viewed as two different branches of research. Texts –or other forms of verbatim– are usually considered to be the domain of qualitative research, however, large bodies of text mandate the use of quantitative methods.

We therefore strongly feel that automated methods ultimately hold the potential to accelerate psychotherapy research by enabling the investigation of therapy models within and across treatments, groups and settings. Automated methods can thus help gain insights in –for example– innovative moments, or the development of therapeutic change processes over time. Interpretation of these research findings will always require human interpretation, which inevitably inspires new research questions.

## Supporting information

S1 Appendix PRISMA statement. The PRISMA 2009 checklist.
(PDF)
